# High-Intensity Interval Training Performed by Young Athletes: A Systematic Review and Meta-Analysis

**DOI:** 10.3389/fphys.2018.01012

**Published:** 2018-07-27

**Authors:** Florian Azad Engel, Alexander Ackermann, Hamdi Chtourou, Billy Sperlich

**Affiliations:** ^1^Department Movement and Training Science, Institute of Sport and Sport Science, Heidelberg University, Heidelberg, Germany; ^2^High Institute of Sport and Physical Education of Sfax, University of Sfax, Sfax, Tunisia; ^3^Department of Sport Science, Integrative and Experimental Training Science, Würzburg University, Würzburg, Germany

**Keywords:** adolescents, physical fitness, aerobic training, peak oxygen uptake, training intensity

## Abstract

**Background:** High-intensity interval training (HIIT) is as a time-efficient alternative to moderate- or low-intensity continuous exercise for improving variables related to endurance and anaerobic performance in young and adolescent athletes.

**Objectives:** To assess original research about enhancement of endurance and anaerobic exercise performance in young and adolescent athletes performing HIIT.

**Method:** Relevant articles published in peer-reviewed journals were retrieved from the electronic databases PubMed and SPORTDiscus in December 2017. Inclusion criteria were: (i) controlled trials (HIIT vs. alternative training protocol) with pre-post design; (ii) healthy young athletes (≤18 years); (iii) assessing variables related to endurance and exercise performance. Hedges' g effect size (ES), and associated 95% confidence intervals were calculated for comparison of any outcome between experimental (HIIT) and alternative training protocol.

**Results:** Twenty four studies, involving 577 athletes (mean age: 15.5 ± 2.2 years), were included in this review. HIIT exerted no or small positive mean ES on peak oxygen uptake (VO_2peak_), running performance, repeated sprint ability, jumping performance and submaximal heart rate. Although the mean ES for changes in VO_2peak_ with HIIT is small (mean g = 0.10±0.28), the average increase in VO_2peak_ from pre to post HIIT-interventions were 7.2 ± 6.9% vs. 4.3 ± 6.9% with any other alternative intervention. HIIT largely and positively affected running speed and oxygen consumption at various lactate- or ventilatory-based thresholds, as well as for sprint running performance. Calculations showed negative mean ES for change-of-direction ability (large), and peak blood lactate concentrations (small). Mean duration per training session for HIIT was shorter than for control interventions (28 ± 15 min vs. 38 ± 24 min).

**Conclusion:** The present findings suggest that young athletes performing HIIT may improve certain important variables related to aerobic, as well as anaerobic, performance. With HIIT, most variables related to endurance improved to a higher extent, compared to alternative training protocols. However, based on ES, HIIT did not show clear superiority to the alternative training protocols. Nevertheless, young athletes may benefit from HIIT as it requires less time per training session leaving more time for training sport specific skills.

## Introduction

High-intensity interval training (HIIT) embraces a variety of interval protocols with varying duration and interspersed recovery breaks involving (i) “repeated sprint training” (RST) with sprints of ~3–7 s duration, interspersed with recovery periods of less than 60 s, (ii) “sprint interval training” (SIT) with ~30 s all-out sprints, and 2–4 min of passive recovery periods, and (iii) HIIT with either short (<45 s) or long (2–4 min) interval durations (Buchheit and Laursen, 2013). Depending on the intensity and duration of the exercise, as well as the recovery and the number of repetitions and sets (Buchheit and Laursen, 2013), HIIT protocols stimulate processes involving the transport and utilization of oxygen, thereby stimulating the enhancement of peak oxygen uptake (VO_2peak_) in adults (Laursen and Jenkins, 2002).

Nowadays, HIIT has become popular for improving variables related to endurance performance among multiple populations, including adult endurance athletes (Kilen et al., 2014; Stöggl and Sperlich, 2014; Stöggl and Björklund, 2017), team sports (Helgerud et al., 2011; Purkhús et al., 2016) and other individual sport events (Bonato et al., 2015; Fernandez-Fernandez et al., 2015; Monks et al., 2017). HIIT is also recommended for improving endurance in moderately trained individuals (Helgerud et al., 2007), sedentary adults (Burgomaster et al., 2008), and in connection with diseases (Meyer et al., 2013; Ellingsen et al., 2017).

In contrast, HIIT performed by children and adolescents is significantly less investigated than HIIT performed by adults. Some investigations in young and adolescent athletes evidence increased VO_2peak_ (Harrison et al., 2015a; Fernandez-Fernandez et al., 2017), shuttle run performance (Buchheit et al., 2008, 2009), sprint (Siegler et al., 2003; Sperlich et al., 2011) as well as repeated sprint (Buchheit et al., 2009) and jump performance (Tønnessen et al., 2011) in connection with HIIT. Overall, a systematic review summarizing the physiological effects of variables on aerobic and anaerobic parameters, as well as sport specific performance in young athletes, is missing. Recent reviews about HIIT and children focus on cardio-respiratory fitness and health-related fitness in children and adolescents (Costigan et al., 2015; García-Hermoso et al., 2016; Bond et al., 2017; Eddolls et al., 2017; Thivel et al., 2018). The reviews whether trained, sedentary and obese (Costigan et al., 2015; Eddolls et al., 2017) or analyzed exclusively obese children (García-Hermoso et al., 2016; Bond et al., 2017; Thivel et al., 2018) to identify the responses of HIIT compared to an alternative training protocol. No review so far has analyzed the various adaptation of HIIT exclusively in young athletes which is important since the adaptation to HIIT may be different in athletes compared to diseased or untrained children.

The purpose of this review is: (i) to summarize and analyse the effects of various intensities, number, and duration of intervals and recovery periods of various HIIT interventions, in contrast to other control interventions, and (ii) to provide evidence-based recommendations for the application of HIIT in young and adolescent athletes.

As a time-efficient training program, HIIT could play an important role in youth athletic development, providing more time for the enhancement of other important skills, such as coordinative skills, technique, tactics, speed, power, strength and many more.

## Methods

### Data sources and literature searching

A systematic review was conducted applying the established guidelines of PRISMA statement (Liberati et al., 2009). A comprehensive computerized search of the electronic databases PubMed (National Center for Biotechnology Information, www.ncbi.nlm.nih.gov/pubmed) and SPORTDiscus (EBSCO, www.ebsco.com/products/research-databases/sportdiscus) was performed during December 2017, with no restriction for the publication year. We employed the following MeSH terms: high intensity interval training OR high intensity training OR intensive interval training OR functional high intensity training OR high intensity circuit training OR aerobic interval training OR sprint interval training OR repeated sprint training OR intensive exercise AND young athletes OR adolescent athletes OR teen athletes OR junior athletes OR children athletes OR children OR adolescents. These strings were further limited to original research studies published in peer-reviewed journals written in English. The titles and abstracts of identified articles in the search process were assessed for inclusion criteria first. Subsequently, full-text articles were retrieved and assessed for inclusion criteria. In addition, reference lists of the identified articles were examined manually for additional relevant titles.

### Inclusion and exclusion criteria

Studies were considered eligible according to the following criteria: (1) prescribed HIIT [e.g., ≥90% of maximal oxygen uptake (Buchheit and Laursen, 2013)], 90–95% peak heart rate (Sperlich et al., 2011) or (supra)maximal interval sprinting (Laursen and Jenkins, 2002); (2) involving children and/or adolescents of any sport (≤18 years; male and female) with performance-related fitness outcomes, sport specific performance, or physiological performance parameters; (3) intervention duration ≥4 weeks or HIIT micro-cycle of ~5–14 days, as defined by Wahl et al. (2013) and Meckel et al. (2014); (4) inclusion of a passive control or alternative training group, including low-intensity exercise, sport-specific training, or other forms of exercise as a control group. Studies were exclusively limited to those involving young athletes, defined to one of the following criteria: *superior athletic talent, undergo specialized training, receive expert coaching, exposure to early competition*” (Armstrong and McManus, 2011). Studies with patients (e.g., obesity, diabetes mellitus, or asthma) and studies involving solely strength training were excluded. Conference abstracts, dissertations, theses and articles published in non-peer-reviewed journals were not included.

### Study selection, data extraction, and quality assessment

In the present analysis, we only included data from investigations providing mean values and measures of variability either published or obtained from the authors. In some cases, the mean and measures of variability were extrapolated from the figures (Faude et al., 2008; Impellizzeri et al., 2008; Sandbakk et al., 2013).

Each study meeting the inclusion criteria was also evaluated by two independent reviewers (FAE and BS) according to the Physiotherapy Evidence Database (PEDro) scale (Sherrington et al., 2000; Olivo et al., 2008) for the methodological quality assessment of the original research studies. We applied the 11-item PEDro scale as described in detail elsewhere (Physiotherapy Evidence Database, 2018). Briefly, a “yes”-answer to one of the 11 questions adds one point, and “no” 0 points with 11-points reflecting greatest study quality. Among others, the PEDro scale comprises following questions: (i) Were the participants randomly allocated to groups?; (ii) Were the groups similar at baseline regarding the most important prognostic indicators?; (iii) Did all participants for whom outcome measures were available received the treatment or control condition as allocated?; (iv) Are the results of between-group statistical comparisons reported for the primary outcome? This approach has been applied previously in systematic reviews to assess methodological quality (Lopez et al., 2009; McDermott et al., 2009; Hart et al., 2010).

### Statistical analyses

Effect sizes (ES) (Hedges' g) and 95% confidence intervals [the difference between the mean values for the experimental and alternative training protocol, divided by the average standard deviation for both groups (Glass, 1977) were calculated]. To optimize the calculation of ES, and estimate the standard deviation for Hedges' g, the standard deviations of the experimental and alternative training protocol groups at baseline were pooled (Wasserman et al., 1988). In accordance with standard practice, the ES values obtained were defined as <0.40 = small, 0.40–0.70 = moderate and >0.70 = large (Higgins and Green, 2011). Heterogeneity across the included studies was assessed using I^2^ calculations (Higgins and Green, 2011).

## Results

### General characteristics of the studies analyzed

**Figure 1:**
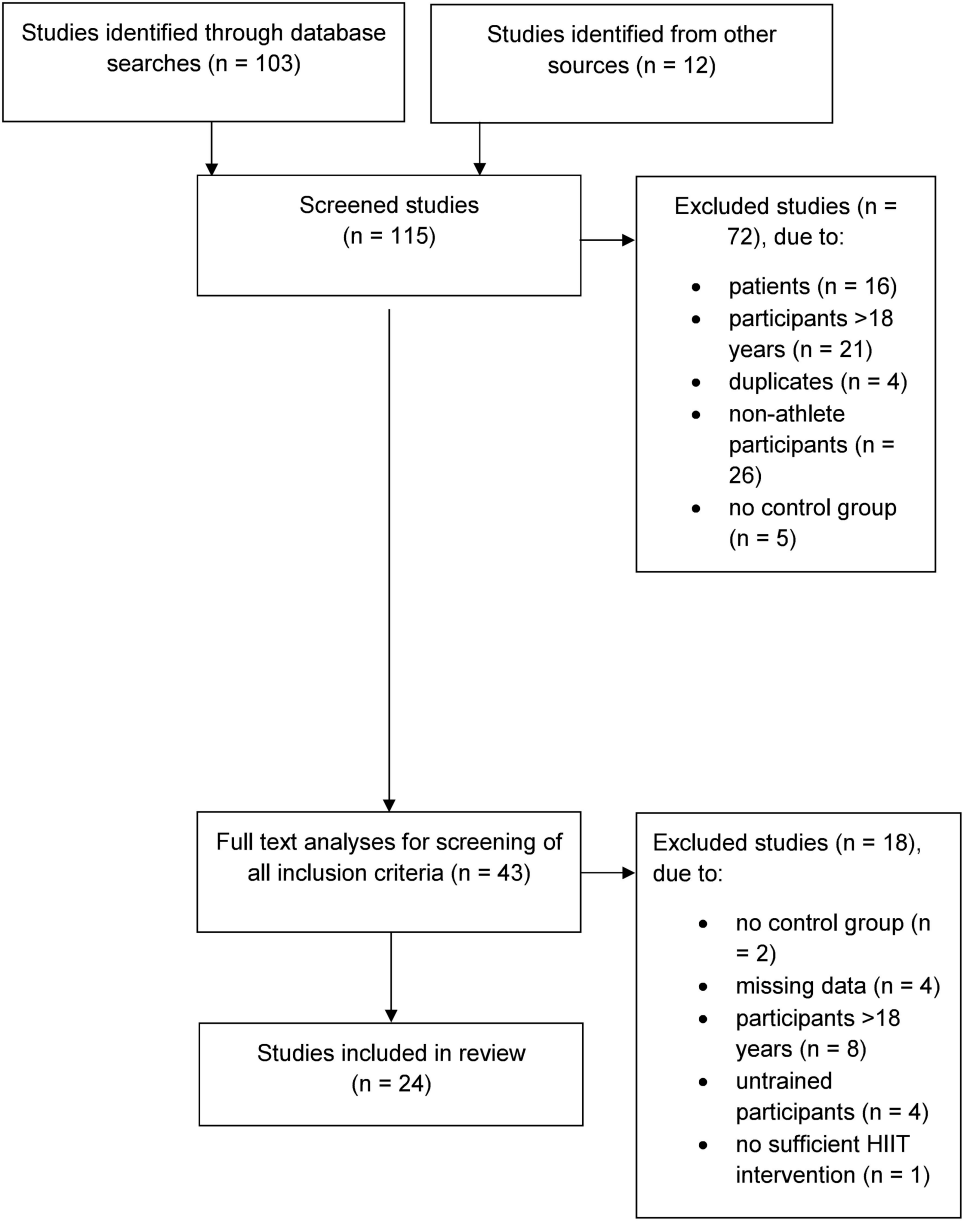
Process of study selection from initial identification to inclusion.

Of the 115 studies initially identified, 24, published between 2001 and 2017, were included in this review (Figure 1). Their average PEDro score was 7.9 (range: 5–9). The detailed characteristics of the 24 analyzed studies are summarized in Table 1.

**Table 1:** Summary of studies included in the present systematic review.

**Authors**	**Participants [n], sex /sport/age [yrs]**	**Sessions [n]**	**Duration [wk]**	**Initial VO_2peak/max_ [ml·min^−1^·kg^−1^ or l/min^−1^]**	**Intensity**	**Number & duration of intervals**	**Duration & intensity rest [work:rest ratio]**	**Post VO_2peak/max_ [ml·min^−1^·kg^−1^ or l/min^−1^]**	**Percentage of change VO_2peak/max_ [%]**	**Main results of HIIT compared to control intervention**
Los Arcos et al., 2015	15 M/Soccer/16	12	6	HIIT						↔ MAS; ↔ CMJ
				n.i.	90–95% HR_max_	3 × 4 min	3 min (50–60% HR_max_) [1:0.75]	n.i.	n.i.	
				SSG						
				n.i.	n.i.	3 × 4 min	3 min	n.i.	n.i.	
Faude et al., 2014	19 M/Soccer/17	8	4	HIIT						↑ IAT; ↓ CMJ; ↔ 5, 10, 30 m Sprint
				n.i.	40% above IAT	2 × (12–15 × 15 s)	10 min/15 s [1:1]	n.i.	n.i.	
				SSG						
				n.i.	n.i.	4 × 4 min	4 min (PR) [1:1]	n.i.	n.i.	
Ferrete et al., 2014	24 n. i./Soccer/9	48	26	Normal soccer training & functional HIIT (squats, jumps, & interval sprints)						↑ YYIERL1; ↑ CMJ; ↑ Sit and Reach Test; ↔ 15 m Sprint
				n.i.	–	–	–	n.i.	n.i.	
				Generic soccer training						
				n.i.	–	–	–	n.i.	n.i.	
Meckel et al., 2014	20 M/Soccer/17	5	5 d	HIIT						↑ VO_2max_; ↓ CMJ; ↓ 250 m Sprint; ↔ 10 m Sprint; ↔ 5 × 10-m run-Agility-test
				51.2 ± 3.6	4:50–3:35 min/km	5–9 × 1,000 m	2:10–3:20 min	54.3 ± 4.2	+ 6.1	
				Continuous low intensity training						
				51.8 ± 3.4	20:25–46:30 min	5,000–9,000 m	–	55.3 ± 5.1	+ 6.8	
Faude et al., 2013	20 M/Soccer/16	12–15	5.5	HIIT						↑ IAT; ↑ HR_max;_ ↓ CMJ; ↓ DJ; ↔ V_max_; ↔ Lac_max_
				n.i.	25–40% above IAT	2 × (12–15 × 15–30 s)	10 min (AR)/ 15–30 s [1:1]	n.i.	n.i.	
				VOT (Fartlek and continuous low intensity training)						
				n.i.	80–95% of IAT	30–60 min	–	n.i.	n.i.	
Safania et al., 2011	20 n. i./Soccer/16	18	6	HIIT						↑ VO_2max_; ↑ W_peak_ RAST; ↑ W_mean_ RAST; ↑ Fatigue RAST
				34 ± 1.4	70–95% HR_max_	4 × 4 min	3 min (50–60% HR_max_) [1:0.75]	43.5 ± 1.4	+ 27.9	
				SSG						
				34.2 ± 1.6	70–95% HR_max_	n.i.	3 min	42.9 ± 1.4	+ 25.4	
Sperlich et al., 2011	19 M/Soccer/13	13	5	HIIT						↑ 1000 m-run; ↑ 20 m, 30 m, 40 m Sprint; ↔ DJ, SJ, CMJ
				55.1 ± 4.9	90–95% HR_max_	4–15 × 30 s-4 min	1–3 min (50–60% HR_max_) [1:1; 1:0.75; 1:2; 2:1]	58.9 ± 4.7	+ 6.9	
				VOT (Fartlek and continuous VOT)						
				55.3 ± 4.3	50–70% HR_max_	6–30 min	–	56.4 ± 3.7	+ 2.0	
Tønnessen et al., 2011	20 M/Soccer/16	10	10	HIIT						↔ Beep-Test; ↑ CMJ; ↑ 20 m, 40 m Sprint; ↑ RSA
				n.i.	95–100% Sprint	3 × (4 × 40 m)	10 min/1 min 30 s	n.i.	n.i.	
				Generic soccer training						
				n.i.	–	–	–	n.i.	n.i.	
Hill-Haas et al., 2009	19 n. i./Soccer/15	14	7	Functional HIIT (intervals, sprints & agility)						↔ VO_2max_; ↑ YYIRTL1; ↔ 5 m, 20 m Sprint; ↔ RSA
				60.2 ± 4.6	–	–	–	61.4 ± 3.5	+ 2.0	
				SSG						
				59.3 ± 4.5	n.i.	2–3 × 6–13 min	1–3 min [1:0.27; 1:0.22; 6:1; 1:0.18; 1:0.15; 5:1; 7:1]	58.9 ± 5.5	– 0.7	
Impellizzeri et al., 2008	21 n. i./Soccer/18	11	4	HIIT						↑ VO_2max_; ↑ passing ability
				~56.6	90–95% HR_max_	4 × 4 min	3 min (AR) [1:0.75]	~ 58.9	~+4	
				Control group (TT)						
				~57.7	–	–	–	~ 57	~−1.2	
Impellizzeri et al., 2006	29 n. i./Soccer/17	16	8	HIIT						↑ VO_2max_; ↑ LT; ↑ RE; ↑ distance covered during soccer match
				59.7 ± 4.1	90–95% HR_max_	4 × 4 min	3 min (60–70% HR_max_) [1:0.75]	60.2 ± 3.9	+ 0.8	
				SSG						
				61.4 ± 4.6	90–95% HR_max_	4 × 4 min	3 min (60–70% HR_max_) [1:0.75]	61.8 ± 4.5	+ 0.7	
Siegler et al., 2003	34 F/Soccer/16	30	10	Functional HIIT (sprinting & jumping)						↑ SRT; ↑ 20 m Sprint; ↑ fat free mass; ↓ body fat; ↔ W_peak_ in WANT
				n.i.	100%	3–5 × 4–6 Reps.	n.i.	n.i.	n.i.	
				VOT						
				n.i.	–	–	–	n.i.	n.i.	
Helgerud et al., 2001	19 M/Soccer/18	16	8	HIIT						↑ VO_2max_; ↑ LT; ↑ RE; ↑ performance parameters in soccer match
				58.1 ± 4.5	90–95% HR_max_	4 × 4 min	3 min (50–60% HR_max_) [1:0.75]	64.3 ± 3.9	+ 10.7	
				Control group (TT)						
				58.4 ± 4.3	–	–	–	59.5 ± 4.4	+ 1.9	
Buchheit et al., 2009	32 (*M* = 16, *F* = 16) /Handball/15	20	10	HIIT						↔ CMJ; ↔ 10 m Sprint; ↑ RSA; ↑ V_IFT_
				n.i.	95% V_IFT_	12–24 × 15 s	15 s (PR) [1:1]	n.i.	n.i.	
				SSG						
				n.i.	n.i.	n.i.	n.i.	n.i.	n.i.	
Buchheit et al., 2008	15 M/Handball/16	15	9	HIIT						↑ CMJ; ↑ V_IFT_; ↑ RSA; ↑%HR_6peak_; ↔ 10 m Sprint; ↔%HR_6mean_
				n.i.	90–95% V_IFT_	9–24 × 15–20 s	15–20 s (PR) [1:1]	n.i.	n.i.	
				SIT						
				n.i.	100–120% V_IFT_	1–3 × (5–6 × 2 × 15–20 m)	2 min (PR)/14–23 s (45% V_IFT_) or PR	n.i.	n.i.	
Delextrat and Martinez, 2014	18 M/Basketball/16	12	6	HIIT						↑ V_IFT_; ↔ RSA; ↔ defensive agility; ↑ offensive agility
				n.i.	90% HR_max_/ 95% V_IFT_	8–13 × 15 s	15 s (AR) [1:1]	n.i.	n.i.	
				SSG						
				n.i.	90% HR_max_	2 × (2–3 × 3–4 min)	PR	n.i.	n.i.	
Harrison et al., 2015a	21 M/Field Hockey and Rugby/14	12	6	HIIT + S						↑ VO_2max_; ↑ V_IFT_; ↔ 5m, 20m Sprint
				55.9 ± 2.5	*HIIT* 90–95% V_IFT_ *SSG* n.i.	*HIIT* 2 × (16-22 × 15 s) *SSG* 16–24 min	*HIIT* 3 min (PR)/15 s (PR) [1:1] *SSG* –	59.0 ± 2.3	+ 5.5	
				SSG						
				55.9 ± 3.0	n.i.	16–24 min or 2 × 8–11 min	3 min (PR) [1:0.38; 1:0.3; 1:0.27]	57.1 ± 3.5	+ 1.6	
Fernandez-Fernandez et al., 2017	17 n.i./Tennis/ 15	16	8	HIIT + TT						↑ VO_2max_; ↑ V_IFT_; ↔ CMJ; ↔ 5, 10, 20m Sprint; ↔ 505 Agility test
				56.2 ± 3.1	*HIIT* 90–95% V_IFT_ *TT* high intensity	*HIIT* 2 × (16 × 15 s) *TT* 2 × 8 min	*HIIT* 3 min(PR)/15 s [1:1] *TT* 3 min [1:0.38]	59.7 ± 3.3	+ 4.2	
				TT						
				56.1 ± 2.2	high intensity	2 × 8 min	3 min [1:0.38]	57.3 ± 2.1	+ 2.4	
Sandbakk et al., 2013	21 (*M* = 12, *F* = 9) /Cross-country Ski/18	16	8	HIIT with short interval						↑ VO_2max_; ↑ VO_2_VT in% of VO_2max_; ↑ 7-km uphill run; ↑ rollerski
				~67.0	95% HR_max_	2–4 min	n.i.	~69.0	+ 3.5	
				HIIT with long intervals						
				~67.0	91% HR_max_	5–10 min	n.i.	~71.0	+ 3.7	
				Continuous low intensity training						
				~68.0	60–74% HR_max_	1.5–3 h	–	~68.0	0~	
Sandbakk et al., 2011	15 (*M* = 10, *F* = 5)/Cross-country Ski/17	n.i.	8	HIIT with long intervals						↑ VO_2max_; ↑ VO_2_VT; ↑ VO_2_VT in% of VO_2max_; ↑ 1.5-km rollerski
				67.5 ± 6.5	85–92% HR_max_	5–10 min	n.i.	70.2 ± 6.8	+ 4.0	
				Continuous low intensity training						
				69.3 ± 7.2	60–74% HR_max_	1.5–3 h	–	70.3 ± 7.3	+ 1.4	
Sperlich et al., 2010	26 (*M* = 13, *F* = 13)/Swimming/10	24	5	HIIT						↑ Lac_max_; ↑ LEN; ↑ T_2000m_; ↔ T_100m_; ↔ VT
				39.9 ± 9.1	92% PB	variably	variably	44.5 ± 7.4	+ 11.5	
				VOT						
				39.4 ± 9.7	85% PB	variably	variably	43.1 ± 6.7	+ 9.4	
Faude et al., 2008	10 (*M* = 6, *F* = 4)/Swimming/16	24	4	HIIT						↑ IAT; ↔ T_100m_; ↔ T_400m_
				n.i.	30.8% above IAT	variably	PR & AR	n.i.	n.i.	
				VOT						
				n.i.	23.3% above IAT	variably	PR & AR	n.i.	n.i.	
Farley et al., 2016	24 (*M* = 19, *F* = 5)/Surfing/14	10	5	HIIT						↑ performance 400 m paddle test; ↔ performance repeat-sprint paddle test; ↑ Fatigue-Index
				n.i.	120% MAS	2–3 × (5–6 × 30 s)	2 min/30 s [1:1]	n.i.	n.i.	
				SIT						
				n.i.	all out	3–6 × (5–8 × 10 s)	2 min/30 s [1:3]	n.i.	n.i.	
Breil et al., 2010	21 (*M* = 15, *F* = 6)/Alpin-Ski/17	15	1.5	HIIT						↑ VO_2max_; ↑ PPO; ↑ VT; ↑ High Box Jump; ↔ BJ90; ↑ La_peak_
				53.0 ± 4.6	90–95% HR_max_	4 × 4 min	3 min (AR) [1:0.75]	56.2 ± 5.1	+ 6.0	
				Generic training						
				52.9 ± 6.3	–	–	–	54.4 ± 7.0	+ 2.8	

*Presented studies conducted controlled HIIT interventions with young and adolescent athletes. ↑, significant positive effect; ↓, significant negative effect; ↔, no significant effect; ↑↓, contradictory results: positive, as well as negative effects; %HR6mean, mean heart rate during 6-min submaximal exercise test; %HR6peak, peak heart rate during 6 min submaximal exercise test; AR, active rest; BJ90, 90-Second Box Jump; CMJ, Counter Movement Jump; DJ, Drop Jump; DOMS, delayed onset muscle soreness; F, female; HR, heart rate; IAT, Individual anaerobic threshold; Lac_max_, maximum blood lactate concentration; LEN, scoring system for swimming performance of European Swimming League; LT, lactate threshold; M, male; MAS, maximum aerobic speed; MB5, Multiple 5-Bounds Test; n.i., not indicated; PB, Personal best; PPO, Peak Power Output in incremental cycling test; PR, passive rest; RAST, Running-based anaerobic sprint test; Reps, Repetitions; RE, running economy; RSA, Repeated Sprint Ability; SIT, Sprint interval training; SJ, Squat Jump; SRT, Shuttle Run Test; SSG, Small-sided game; T100, time for 100 m swimming; T400, time for 400 m swimming; T2000, time for 2,000 m swimming; TS, Training session; TT, Technique training; V_IFT_, maximum velocity in 30–15 Intermittent Fitness Test; V_max_, maximum velocity in incremental steptest; VO_2max_, maximal oxygen uptake; VO_2peak_, highest measured oxygen uptake; VO2VT, oxygen consumption at ventilatory threshold; VOT, Volumen oriented training at low intensities; VT, Ventilatory threshold; WANT, Wingate Anaerobic Test; W_mean_, Mean Power output; W_peak_, Peak Power output; YYIERL1, Yo-Yo Intermittent Endurance Run: Level 1; YYIRTL1, Yo-Yo Intermittent Recovery Test Level 1*.

The 24 analyzed studies involved a total of 577 participants aged 9–18 years (mean age: 15.5 ± 2.2 years). Wherein 287 participants (*n* = 24 studies) completed a form of HIIT, and 290 participants (*n* = 24 studies) completed an alternative training protocol (i.e., small-sided games, high-intensity endurance training [~91% HR_max_ (Sandbakk et al., 2013)], low-intensity continuous endurance training [60–74% HR_max_ (Sandbakk et al., 2011, 2013), 80–95% of individual anaerobic threshold (Faude et al., 2013), 50–70% HR_max_ (Sperlich et al., 2011)], Fartlek method running [50–70% HR_max_ (Sperlich et al., 2011)], sport-specific technique training or ordinary sport specific training) as a control condition. None of the analyzed studies included a control group without any kind of exercise.

The mean sample size was *n* = 24.0 ± 9.2 (mean ± SD; range: 15–52) participants. Eleven studies included boys (Helgerud et al., 2001; Impellizzeri et al., 2006; Buchheit et al., 2008; Sperlich et al., 2011; Tønnessen et al., 2011; Faude et al., 2013, 2014; Delextrat and Martinez, 2014; Meckel et al., 2014; Harrison et al., 2015a; Los Arcos et al., 2015), seven studies included boys and girls (Faude et al., 2008; Buchheit et al., 2009; Breil et al., 2010; Sperlich et al., 2010; Sandbakk et al., 2011, 2013; Farley et al., 2016), one study included exclusively girls (Siegler et al., 2003) and six studies (Impellizzeri et al., 2006, 2008; Hill-Haas et al., 2009; Safania et al., 2011; Ferrete et al., 2014; Fernandez-Fernandez et al., 2017) provided no explicit information about the participant's sex.

**Figure 2:**
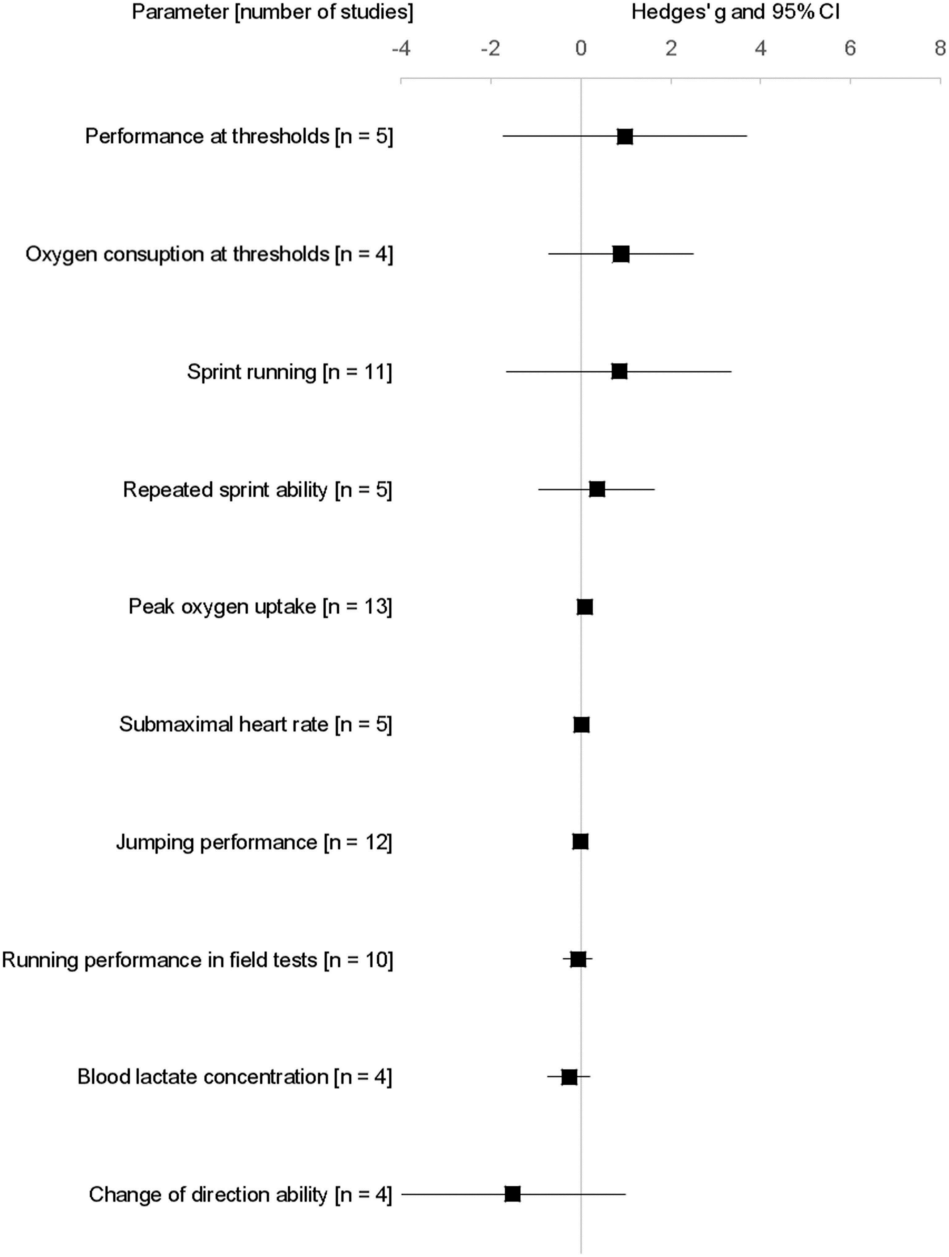
Hedges' g effect sizes (square) and associated 95% confidence interval (lines) of the application of High intensity interval training according to various performance indicators. n, number of studies.

HIIT studies were conducted in following sports: Soccer (*n* = 13), cross-country skiing (*n* = 2), handball (*n* = 2), swimming (*n* = 2), alpine ski (*n* = 1), basketball (*n* = 1), field hockey and rugby (*n* = 1), surfing (*n* = 1), and tennis (*n* = 1). The participants' initial VO_2peak_ ranged from 34.0 to 69.3 ml·min^−1^·kg^−1^ (mean: 54.1 ± 9.0 ml·min^−1^·kg^−1^). Wherein 13 out of 24 studies determined VO_2peak_ of participants, 11 studies with direct measurement (Helgerud et al., 2001; Impellizzeri et al., 2006, 2008; Hill-Haas et al., 2009; Breil et al., 2010; Sperlich et al., 2010, 2011; Sandbakk et al., 2011, 2013; Harrison et al., 2015a; Fernandez-Fernandez et al., 2017) and two studies with indirect measurement (Safania et al., 2011; Meckel et al., 2014). The various ES calculations are illustrated in Figure 2.

### Protocols and periodization of HIIT interventions

The 24 studies applied various HIIT or SIT involving a variety of intensities and durations, as well as various work-to-rest ratios (Table 1). Most of the studies performed HIIT as running-based protocols. Some studies applied functional HIIT programs, consisting of squats and jumps, as well as other exercises and sprints performed according to typical HIIT protocols (Siegler et al., 2003; Hill-Haas et al., 2009; Ferrete et al., 2014).

The intensity corresponding to 90–95% of maximum heart rate (HR_max_) (Helgerud et al., 2001; Impellizzeri et al., 2006, 2008; Breil et al., 2010; Safania et al., 2011; Sandbakk et al., 2011, 2013; Sperlich et al., 2011; Delextrat and Martinez, 2014; Los Arcos et al., 2015) as well as 90–95% of maximum running velocity (v_max_) (Buchheit et al., 2008; Delextrat and Martinez, 2014; Harrison et al., 2015a; Fernandez-Fernandez et al., 2017), both derived from incremental step tests (e.g., intermittent fitness test, 30–15 intermittent fitness test) defined the intensity in most HIIT. Furthermore, intensities above the individual anaerobic threshold (Faude et al., 2008, 2013, 2014) were applied as HIIT protocols as well. Additionally, “all out” efforts, representing maximal sprinting speed, (Siegler et al., 2003; Hill-Haas et al., 2009; Ferrete et al., 2014; Farley et al., 2016) defined the intensity of HIIT protocols. The majority of studies monitored training intensities with heart rate monitors (Impellizzeri et al., 2006; Buchheit et al., 2008, 2009; Hill-Haas et al., 2009; Breil et al., 2010; Sandbakk et al., 2011, 2013; Sperlich et al., 2011; Faude et al., 2013, 2014; Delextrat and Martinez, 2014; Fernandez-Fernandez et al., 2015; Harrison et al., 2015a; Los Arcos et al., 2015) or velocity (Faude et al., 2008; Sperlich et al., 2010; Meckel et al., 2014; Farley et al., 2016) to ensure appropriate exercise intensity. Five studies provided no explicit information about training intensity monitoring (Helgerud et al., 2001; Siegler et al., 2003; Impellizzeri et al., 2008; Safania et al., 2011; Tønnessen et al., 2011).

The duration and corresponding intensities of intervals of all studies were as follows: (i) short intervals ~10–15 s, performed with maximal sprinting speed; (ii) medium duration intervals 30 s−2 min, performed with maximal sprinting speed or 90–95% HR_max_ or 90–95% v_max_; (iii) long intervals 4–10 min, performed with 90–95% HR_max_ or 90–95% v_max_.

The intervention period of the 24 studies averaged 7.5 weeks (range: 5 days−26 weeks) with a mean of 2.5 HIIT sessions per week (range: 1–6). Two studies conducted condensed sessions of HIIT employing five (Meckel et al., 2014) and 11 (Breil et al., 2010) days, respectively, of HIIT. The mean duration per HIIT training session was 28 ± 15 min and 38 ± 24 min for the alternative training protocol. The training session duration in seven out of 24 studies could not be assessed due to missing or inaccurate details (Siegler et al., 2003; Impellizzeri et al., 2006; Faude et al., 2008; Breil et al., 2010; Sandbakk et al., 2011; Tønnessen et al., 2011; Ferrete et al., 2014).

### Maximum or peak oxygen uptake

Our analysis revealed that HIIT and SIT has no, or only a small, positive effect [mean g = 0.10 ± 0.28; range: −0.63–0.48 (Helgerud et al., 2001; Impellizzeri et al., 2006, 2008; Hill-Haas et al., 2009; Breil et al., 2010; Sperlich et al., 2010, 2011; Safania et al., 2011; Sandbakk et al., 2011, 2013; Meckel et al., 2014; Harrison et al., 2015a; Fernandez-Fernandez et al., 2017)] on peak oxygen uptake, in comparison to the alternative training protocol (Table 2).

**Table 2:** Statistical analysis of HIIT vs. control intervention (e.g., low intensity continuous endurance training, low intensity interval training, high-intensity endurance training, small sided games, sport specific technique training, or ordinary sport specific training) comparing the response of peak oxygen uptake (VO_2peak_).

			**95% Confidence interval**	
**Study name**	**Unit of VO**_2peak_	**Hedge's g**	**Lower limit**	**Upper limit**
Sandbakk et al., 2011	L·min^−1^	−0.63	−0.83	−0.43
Impellizzeri et al., 2006	L·min^−1^	−0.32	−0.36	−0.27
Breil et al., 2010	L·min^−1^	−0.17	−0.34	−0.01
Impellizzeri et al., 2006	ml·kg^−1^·min^−1^	−0.12	−4.72	4.48
Meckel et al., 2014	ml·kg^−1^·min^−1^	−0.06	−6.70	6.57
Sandbakk et al., 2011	ml·kg^−1^·min^−1^	0.00	−17.07	17.06
Harrison et al., 2015a	ml·kg^−0, 75^·min^−1^	0.02	−40.94	40.98
Sperlich et al., 2010	ml·kg^−1^·min^−1^	0.04	−9.23	9.31
Breil et al., 2010	ml·kg^−1^·min^−1^	0.06	−12.07	12.20
Hill-Haas et al., 2009	ml·kg^−0, 75^·min^−1^	0.09	−33.84	34.02
Hill-Haas et al., 2009	ml·kg^−1^·min^−1^	0.18	−5.76	6.12
Sperlich et al., 2011	ml·kg^−1^·min^−1^	0.19	−5.85	6.22
Impellizzeri et al., 2008	ml·kg^−1^·min^−1^	0.21	−3.48	3.90
Helgerud et al., 2001	ml·kg^−0, 75^·min^−1^	0.24	−31.80	32.28
Harrison et al., 2015a	ml·kg^−1^·min^−1^	0.32	−1.86	2.51
Helgerud et al., 2001	ml·kg^−1^·min^−1^	0.39	−4.90	5.68
Safania et al., 2011	ml·kg^−1^·min^−1^	0.41	−0.20	1.02
Fernandez-Fernandez et al., 2017	ml·kg^−1^·min^−1^	0.48	−1.77	2.73
Helgerud et al., 2001	L·min^−1^	0.48	0.05	0.91
Mean Hedge's g		0.10		

*L·min^−1^–absolute VO_2peak_; ml·kg^−0, 75^·min^−1^–allometric VO_2peak_; ml·kg^−1^·min^−1^–relative VO_2peak_*.

The mean percent increase of VO_2peak_ from pre to post was 7.2 ± 6.9% in the HIIT groups, and 4.3 ± 6.9% in the alternative training protocol groups, with a corresponding absolute increase of 3.5 ± 2.3 ml·min^−1^·kg^−1^ in HIIT vs. 1.7 ± 2.5 ml·min^−1^·kg^−1^ in the alternative training protocol groups. The absolute increase in VO_2peak_ per training session with HIIT was 0.26 ± 0.2 ml·min^−1^·kg^−1^ vs. 0.15 ± 0.2 ml·min^−1^·kg^−1^ in the alternative training protocol groups.

The *I*^2^ analysis indicated moderate heterogeneity (44%).

### Running performance in incremental field tests

HIIT induced a small negative effect [mean g = −0.07 ± 0.45; range: −0.99–0.70 (Buchheit et al., 2008, 2009; Hill-Haas et al., 2009; Tønnessen et al., 2011; Faude et al., 2013, 2014; Delextrat and Martinez, 2014; Ferrete et al., 2014; Harrison et al., 2015a; Los Arcos et al., 2015)] on endurance running performance in various incremental running tests, compared to alternative training interventions (Table 3, Figure 2). The *I*^2^ analysis suggests substantial heterogeneity (60%).

**Table 3:** Statistical analysis of HIIT vs. control intervention (e.g., low intensity continuous endurance training, low intensity interval training, small sided games, sport specific technique training or ordinary sport specific training) comparing the performance in different incremental field running tests.

			**95% Confidence interval**	
**Study name**	**Parameter**	**Hedge's g**	**Lower limit**	**Upper limit**
Faude et al., 2014	Peak running speed in incremental field test (km·h^−1^)	−0.99	−1.15	−0.83
Buchheit et al., 2009	Final running speed in 30–15 IFT (km·h^−1^)	−0.51	−0.97	−0.05
Faude et al., 2013	Peak running speed in endurance test (km·h^−1^)	−0.50	−0.70	−0.31
Tønnessen et al., 2011	Final level Beep–test (n)	0.00	−0.56	0.56
Ferrete et al., 2014	Distance completed in Yo–Yo Intermittent Endurance Run: Level 1 (m)	0.00	−1.41	1.41
Hill-Haas et al., 2009	Distance completed in Multi–Stage Fitness Test (m)	0.00	−1.19	1.19
Hill-Haas et al., 2009	Distance completed in YoYo Intermittent recovery Test Level 1 (m)	0.01	−2.91	2.91
Delextrat and Martinez, 2014	Final running speed in 30–15 IFT (km·h^−1^)	0.09	−0.40	0.58
Buchheit et al., 2008	Final running speed in 30–15 IFT (km·h^−1^)	0.09	−0.92	1.11
Los Arcos et al., 2015	Final running speed in UM–TT (km·h^−1^)	0.32	0.02	0.62
Harrison et al., 2015a	Final running speed in 30–15 IFT (km·h^−1^)	0.70	0.43	0.97
Mean Hedge's g		−0.07		

*30–15 IFT–30–15 Intermittent Fitness Test. UM–TT – Université de Montréal Tract Test*.

### Performance at various thresholds

HIIT induced a large positive effect on running velocities and performance at various thresholds [mean g = 0.97 ± 3.39; range: −1.35–7.81 (Helgerud et al., 2001; Impellizzeri et al., 2006; Breil et al., 2010; Faude et al., 2013; Los Arcos et al., 2015)] (Figure 2), whereas the ES showed a large variation, with one study exhibiting a large positive ES of 7.8 (Helgerud et al., 2001), two studies revealing no effect [ES = 0.00 (Los Arcos et al., 2015)] and 0.01 (Breil et al., 2010), and two studies showing a negative ES of −0.65 (Faude et al., 2013) and −1.35 (Impellizzeri et al., 2006).

The *I*^2^ analysis indicates a moderate heterogeneity (42%).

### Repeated sprint ability

In the five investigations that evaluated responses of HIIT on the repeated sprint ability of young athletes, a small positive ES was observed [mean g = 0.35 ± 1.48; range: −1.27–2.69 (Buchheit et al., 2008, 2009; Hill-Haas et al., 2009; Tønnessen et al., 2011; Delextrat and Martinez, 2014)] (Table 4, Figure 2).

**Table 4:** Statistical analysis of HIIT vs. control intervention (e.g., low intensity continuous endurance training, low intensity interval training, small sided games, sport specific technique training or ordinary sport specific training) comparing performance in repeated sprint tests.

			**95% Confidence interval**	
**Study name**	**Parameter of RSA test**	**Hedge's g**	**Lower limit**	**Upper limit**
Buchheit et al., 2009	Mean sprint time 6 × 2 × 15 m (s)	−1.27	−1.24	−1.30
Hill-Haas et al., 2009	Total sprint time 12 × 20 m (s)	−0.20	−0.45	0.85
Buchheit et al., 2008	Mean sprint time 6 × 2 × 15 m (s)	−0.12	0.24	−0.01
Delextrat and Martinez, 2014	Total sprint time 6 × 20 m (s)	0.67	0.45	1.79
Tønnessen et al., 2011	Mean sprint time 10 × 40 m	2.69	2.68	2.70
Mean Hedge's g		0.35		

*RSA, Repeated Sprint Ability*.

The *I*^2^ analysis indicates a moderate heterogeneity (40%).

### Change of direction ability

HIIT exerted a large negative effect [mean g = −1.51 ± 3.8; range: −6.69–1.91 (Buchheit et al., 2008; Faude et al., 2014; Meckel et al., 2014; Fernandez-Fernandez et al., 2017)] on change of direction ability (Figure 2), which was assessed in four of the studies with four separate tests (505 agility test; 4 × 5 m shuttle run; 5 × 10 m run agility test; change in direction run).

The *I*^2^ calculation suggests minor heterogeneity (25%).

### Sprint running performance

Eleven studies evaluated the impact of HIIT on sprint running performance. The analyses of 20 sprint running times from 5–40 m demonstrated that HIIT induces large positive effects on sprint running performance [mean g = 0.85 ± 5.95; range:−13.04–14.32 (Siegler et al., 2003; Buchheit et al., 2008, 2009; Hill-Haas et al., 2009)] (Figure 2; Sperlich et al., 2011; Tønnessen et al., 2011; Faude et al., 2014; Ferrete et al., 2014; Meckel et al., 2014; Harrison et al., 2015a; Fernandez-Fernandez et al., 2017).

*I*^2^ statistic revealed a minor heterogeneity (28%).

### Jumping performance

In the 12 studies investigating the responses of HIIT on jump performance (counter movement jump, drop jump, and squat jump) of trained children and adolescents, a small ES on jumping height was observed [mean g = 0.00 ± 0.15; range: −0.15–0.54 (Buchheit et al., 2008, 2009; Breil et al., 2010; Sperlich et al., 2011; Tønnessen et al., 2011; Faude et al., 2013, 2014; Ferrete et al., 2014; Meckel et al., 2014; Harrison et al., 2015a; Los Arcos et al., 2015; Fernandez-Fernandez et al., 2017] (Figure 2).

The *I*^2^ statistic indicated no heterogeneity (0%).

### Blood lactate concentration

In most cases peak blood lactate concentrations during ramp testing, or incremental step testing, were not affected following HIIT interventions [mean g = −0.27 ± 0.47; range: −0.93–0.03 (Helgerud et al., 2001; Breil et al., 2010; Faude et al., 2013, 2014)] (Figure 2), although one study reported a large negative effect on this parameter (increased LA_peak_) in a ramp test [g = −0.93 (Breil et al., 2010)].

The *I*^2^ statistic indicated no heterogeneity (0%).

### Oxygen consumption at various thresholds

Four studies assessed the oxygen consumption at blood lactate threshold and ventilatory threshold 1 and 2, both before and following a period of HIIT, and the analyses revealed a large ES [mean g = 0.89 ± 2.17; range: −0.25–5.80 (Helgerud et al., 2001; Impellizzeri et al., 2006; Breil et al., 2010; Sandbakk et al., 2011)] (Figure 2).

*I*^2^ analysis indicated a moderate heterogeneity (40%).

### Submaximal heart rate

The submaximal heart rate was assessed in five studies, demonstrating a small effect in reducing submaximal heart rate after a period of HIIT [mean g = 0.02 ± 0.07; range: −0.05–0.12 (Helgerud et al., 2001; Impellizzeri et al., 2006; Breil et al., 2010; Faude et al., 2013, 2014)] (Figure 2).

*I*^2^ analysis indicated considerable heterogeneity (99%).

## Discussion

The present systematic review revealed a growing body of literature (from 2001 to present) examining the application of HIIT in young and adolescent athletes from various disciplines.

HIIT seems to be superior, compared to alternative training protocol conditions, for enhancing submaximal endurance performance (running velocities and oxygen uptake at different thresholds), as well as for the enhancement of repeated sprint ability and linear sprint running. However, when compared to other (low- and high-intensity) control interventions, HIIT did not show clear superiority to alternative training regimes for enhancing VO_2peak_ and running performance in incremental tests.

In many sports, VO_2peak_ represents a key component for success (Helgerud et al., 2001; Hoff and Helgerud, 2004; Narazaki et al., 2009; Ben Abdelkrim et al., 2010), and is considered beneficial for sport-specific performance of young competitive players (Harrison et al., 2015b). In recent reviews the large positive ES of HIIT on VO_2peak_, when compared to alternative training programs in adolescents (Costigan et al., 2015) and adults (Bacon et al., 2013) (interval training vs. continuous or combined interval and continuous training), as well as in 18–45 year old adults (Milanović et al., 2015), when compared with no-exercise controls, was demonstrated. Since ES calculation for VO_2peak_ in the present review revealed only a small ES this finding is not in line compared to recent reviews involving untrained adolescents (Costigan et al., 2015) and adults (Bacon et al., 2013; Milanović et al., 2015). In addition to the present ES-based comparison, HIIT exhibited a superior effect on VO_2peak_, the mean percentage of enhancement was 7.2 ± 6.9%, in contrast to 4.3 ± 6.9% in the alternative training protocol interventions. Based on our data a mean increase in VO_2peak_ per training session of 0.26 ± 0.2 ml·min^−1^·kg^−1^ is possible with HIIT compared to 0.15 ± 0.2 ml·min^−1^·kg^−1^ with alternative training. However, the percentage of enhancement in VO_2peak_ following HIIT and alternative protocols, exhibited substantial standard deviations in relation to the mean values indicating a quite substantial variation in the response to HIIT or an alternative training protocol. Furthermore, the small effect size (g = 0.10) reported for VO_2peak_ displays a 95% CI of 0.28, which emphasizes the small effect in comparison to the alternative training protocols. This indicates that HIIT and the completed alternative training regimes improved VO_2peak_ similarly. Considering that all included studies completed an alternative training protocol as a control condition, and in some cases these protocols applied potentially high intensities [e.g., sprint interval training (Buchheit et al., 2008; Farley et al., 2016) and small-sided games (Impellizzeri et al., 2006; Hill-Haas et al., 2009; Safania et al., 2011; Delextrat and Martinez, 2014; Faude et al., 2014; Harrison et al., 2015a; Los Arcos et al., 2015)], it is not surprising that some performance-related parameters of the HIIT groups exhibited partly negative or (to the best) small positive ESs when compared to the control intervention.

Recent systematic reviews examining HIIT in young and adolescent populations revealed large positive effects on cardiorespiratory fitness and/or VO_2peak_ in comparison to alternative training interventions (Costigan et al., 2015; García-Hermoso et al., 2016; Eddolls et al., 2017; Thivel et al., 2018). Whereas, the present review includes only studies with trained children and adolescents exhibiting a rather high baseline VO_2max_ (mean: 54.1 ± 9.0 ml·min^−1^·kg^−1^, range: 34.0–69.3 ml·min^−1^·kg^−^1) with only two studies exhibiting a VO_2max_ < 40 ml·min^−1^·kg^−1^ (Sperlich et al., 2010; Safania et al., 2011). In comparison to the present review, the initial VO_2peak_ in participants of other reviews (Costigan et al., 2015; García-Hermoso et al., 2016; Eddolls et al., 2017; Thivel et al., 2018) were comparably low. Since our analysis involved trained children and adolescents, further adaptations may need more sessions with either longer or more intense exercise when comparison to untrained.

In contrast to the rigorous investigation to the various responses in adults performing HIIT, far less research is available about children and adolescents. In children, changes in VO_2max_ are primarily attributed to increased stroke volume due to increased pre-load, decreased after-load and cardiac enlargement (Nottin et al., 2002). However, in adults, the increase in maximum cardiac output observed after several weeks of endurance training was related to exercise-induced hematological adaptations (Bonne et al., 2014; Montero et al., 2015). In addition to hematological changes, peripheral adaptions targeting oxygen transport, and utilization [e.g., improved capillary and mitochondrial density, improved mitochondria enzyme reactions (Wagner, 1991; Gibala et al., 2012)] also explain increases of VO_2peak_, although not primarily (Montero et al., 2015; Lundby and Jacobs, 2016). Current studies have intensively investigated the responses of HIIT in adults on muscle adaptation (Coffey and Hawley, 2007; Talanian et al., 2007; Little et al., 2011; Metcalfe et al., 2015; MacInnis and Gibala, 2017), however, to the best of our knowledge, there are no studies available which analyze mitochondrial adaptations to HIIT or SIT in children or adolescents.

Regarding fatigue resistance, children perform high intensive (interval) exercise with less absolute and relative power output than adults, however, recovery of performance and physiological parameters is more rapid compared to adults (Hebestreit et al., 1993; Falk and Dotan, 2006; Buchheit et al., 2010a; Engel et al., 2015). Furthermore, children show greater oxidative and lower anaerobic and glycolytic capacities, which is represented in their muscle substrate and enzyme activity levels (Eriksson et al., 1973; Berg et al., 1986; Kaczor et al., 2005). A limited glycolytic capacity and a greater reliance on oxidative metabolism leads to diminished relative and absolute performance parameters during intensive interval exercise, but is suggested as one reason for enhanced recovery (Falk and Dotan, 2006). In addition, VO_2_ kinetics at the onset of exercise (Springer et al., 1991; Williams et al., 2001) is rapid in children, as well as the post-exercise blood lactate elimination (Engel et al., 2015), and heart rate recovery (Buchheit et al., 2010a) following intensive exercise.

### Overreaching

Physically challenging training methods, like SIT and HIIT, require a high level of motivation and confidence (Hardcastle et al., 2014). Even for young athletes, HIIT necessitates a high level of motivation and confidence to sustain the high intensities. Studies of children and adolescents showed equivocal results in respect to perception of HIIT. Adolescent boys (Malik et al., 2017) perceive HIIT as positive, whereas young soccer players enjoyed SSG more than HIIT (Los Arcos et al., 2015), and young swimmers (Sperlich et al., 2010) and soccer players (Sperlich et al., 2011) perceived HIIT as more intense than continuous low intensity endurance training. Generally, the activity patterns of HIIT with bouts of intensive exercise, interspersed with recovery periods, seems to suit children from a psychological perspective. In natural conditions, children (6–10 years) tend to engage in short bursts of intense physical activity, interspersed with varying intervals of low and moderate intensity (Bailey et al., 1995)—an activity pattern similar to HIIT and SIT. However, only a few studies of the present analyses incorporated subjective measures of perceived intensity or enjoyment of HIIT, so it is difficult to judge the influence of HIIT on psychological variables. In addition, only a few studies included objective and subjective measures estimating the current recovery-stress states during HIIT interventions (Faude et al., 2014), showing that HIIT can lead to early signs of fatigue (Faude et al., 2014). Recent studies demonstrate the high cardiorespiratory, metabolic, and hormonal perturbations of a single HIIT session with young athletes (Engel et al., 2014; Kilian et al., 2016). Furthermore, Zinner et al. (2014) showed no habituation of cortisol production in young athletes, following 14 days of HIIT microcycle, which suggests a constant exposure to catabolic hormones of young athletes performing HIIT. Whereas one HIIT session in 14-year old cyclists, led to higher metabolic and cardiorespiratory stress compared to continuous low intensity endurance training; HIIT induces no strong acute catabolic effect, as evidenced by the levels of cortisol, testosterone, and alpha-amylase (Kilian et al., 2016). It is worth noting, however, that the available research on that matter is very limited in number and scope. Considering the high incidence of overtraining in young athletes (Kenttä et al., 2001; Winsley and Matos, 2011), the analyses of recovery-stress states during HIIT interventions, by psychological and physiological methods, would be preferable in evaluating the impact of HIIT on that parameter. Furthermore, in order to prevent staleness and overtraining syndromes, current guidelines (e.g., 1–2 days with no training per week; not more than a weekly increase of 10% in training time, number of repetitions, or total distance; ensure an appropriate off-season) should be respected (Brenner, 2007). Finally, the majority of HIIT studies were conducted over a relatively short periods of time (i.e., 5 days−26 weeks) and the long-term adherence and effects on recovery-stress states are unknown.

### Practical relevance

Several findings (Buchheit et al., 2009; Sperlich et al., 2011; Fernandez-Fernandez et al., 2017; Monks et al., 2017) advocate the time efficiency of HIIT in improving performance and physiological parameters as one of the main advantages of its incorporation in training with young athletes and sedentary individuals (Costigan et al., 2015; García-Hermoso et al., 2016). Likewise, the present review revealed a substantial shorter mean duration of training sessions in the HIIT interventions compared to the control interventions which emphasizes the time-efficiency benefit of HIIT and SIT in the training of young athletes. Exercise time, especially in children, is limited because of other factors, such as school and recreational activities. According to the aforementioned authors, short training sessions with HIIT allow more time for improving sport-specific skills, as well as tactics, which are important components of training in young athletes (Buchheit et al., 2009; Sperlich et al., 2011; Fernandez-Fernandez et al., 2017).

According to present recommendations (Harrison et al., 2015b), aerobic fitness should be developed throughout all development stages of young athletes and not limited to a certain age or maturation period. However, the requirements of high-level youth sports demand the pursuit of multiple aims, which can be difficult with a limited time budget. Consequently, HIIT could be a time-efficient and appropriate training method of enhancing endurance-relevant parameters, since HIIT improves VO_2max_ to a higher extent, compared to other training strategies (7.2 vs. 4.3%), despite differences in training volume. Furthermore, the level of cardiorespiratory fitness is linked to risks of obesity, type 2 diabetes mellitus, and cardiovascular disease (Bouchard et al., 2015). In young athletes, the development of high cardiorespiratory fitness could be a potential protective factor for obesity, type 2 diabetes mellitus and cardiovascular diseases, and could be linked to beneficial health aspects, such as improved cardio-metabolic health.

The majority of analyzed studies were conducted in game-based sports like soccer (*n* = 13 studies), handball (*n* = 2 studies), and basketball (*n* = 1). Game-based sports played by young athletes involve bouts of repetitive short-term high intensity efforts, interspersed with aerobic activities of low to medium intensity (Buchheit et al., 2010b; Harley et al., 2010; Mendez-Villanueva et al., 2013), a pattern which is represented in HIIT.

Within these sports it has been discussed whether sport-specific endurance should be trained, either by playing small-sided games (SSG), or instead, solely as running-based protocols (Halouani et al., 2014). It has been proposed that running protocols are potentially unpleasant for (handball) players (Buchheit et al., 2009), comparatively young players seem to enjoy SSG more than running protocols (Los Arcos et al., 2015). In contrast, male adolescents perceived HIIT as positive (Malik et al., 2017). Moreover, coaches expect to accomplish the optimum training benefits, when training represents the specific movement patterns and physiological demands of the sport (Halouani et al., 2014). Several studies demonstrated that SSG represent a sufficient training modality to enhance aerobic capacity (Impellizzeri et al., 2006; Hill-Haas et al., 2009; Faude et al., 2014; Harrison et al., 2015a) and important anaerobic performance parameters (Faude et al., 2014; Harrison et al., 2015a) in young and adolescent team sports athletes.

Furthermore, SSG are supposed to improve sport-specific skills, movements and tactics (Halouani et al., 2014), and enjoyment during SSG is greater, compared to HIIT (Los Arcos et al., 2015). Based on our findings we may conclude that small-sided games are a sufficient training tool to increase the aerobic and anaerobic performance parameters (Buchheit et al., 2009) of young and adolescent team sport athletes, whereas our analyses revealed that HIIT still induces a higher elevation of VO_2max_ than SSG in young athletes.

Considering the improvement of non-endurance related parameters with HIIT, our analyses revealed that HIIT seems to be a sufficient stimulus to improve anaerobic performance parameters, e.g., repeated sprint ability and linear sprint running performance. Within the original research, studies showed significant improvements in anaerobic performance parameters like sprinting (Siegler et al., 2003; Sperlich et al., 2011; Tønnessen et al., 2011), repeated sprinting (Buchheit et al., 2008, 2009; Tønnessen et al., 2011), and jumping (Buchheit et al., 2008; Tønnessen et al., 2011; Ferrete et al., 2014), which may demonstrate potential cross-effects of HIIT on anaerobic parameters.

### Limitations

To emphasize the quality of the present evidence (i.e., assessing the risk of bias) we would like to point out, that most of the analyzed parameters exhibited no (Jumping performance, blood lactate concentration), minor (Change of direction ability, sprint running performance) or a moderate (VO_2peak_, performance at thresholds, repeated sprint ability, oxygen consumption at various thresholds) heterogeinity. Only two parameters revealed a substantial heterogeneity (running performance, submaximal heart rate), and the mean PEDro score of the included studies with 7.9 (range: 5–9) was high. Taken together this indicates the relatively low probability of risk of bias in the present review.

Due to the range of different HIIT protocols including intensity, duration, frequency and rest durations as well as different control conditions (Table 1) we cannot with certainty determine an optimal HIIT protocol for youth athletes. Particularly in high level youth sport it will be important to individualize the HIIT programm parameters [interval intensity and duration, rest intensity and duration, exercise modality, number of repetitions, number of series, between-series recovery duration and intensity (Buchheit and Laursen, 2013)] of HIIT protocols in order to achieve optimal stimulus for adaptations.

The present review contains a large participnats' range of age (9–18 years), which potentially limits interpretation and generalization of findings. More research is warranted in analyzing differences of impact from HIIT on performance parameters between prepubescent, pubescent and postpubescent athletes.

A general criticism of conducting meta-analyses of studies examing performance parameters following a training intervention is expressed by Gentil et al. (2017). The authors emphasize that variablity among studies may results from many confounding factors including: (i) determination of training intensities, (ii) inadequate supervision of intervention; (iii) different training modes and (iv) assessment of performance parameters in pre- and posttests with different test procedures (Gentil et al., 2017). Although Gentil et al. (2017) are referring to specific problems in the field of strength training, similar problems could appear for the present meta-analysis.

The mean sample size of studies reviewed was *n* = 24.0 ± 9.2 (range: 15–52), which represents a typical sample size with HIIT studies. Whereas, some studies included very small sample sizes ≤15 participants (Buchheit et al., 2008; Faude et al., 2008; Sandbakk et al., 2011; Los Arcos et al., 2015). However, the total number of *n* = 24 studies and 577 participants included in the present review represents a sufficient number for a systematic review (Valentine et al., 2010) to provide an adequate overview of adapation to HIIT in young athletes.

Considering the effects of HIIT between male and female athletes, no conclusions could be drawn as none of the studies included in this review provided sufficient results between gender. Only one study provided exclusively results for female athletes (Siegler et al., 2003). This demonstrates that the growing and relevant population of girl athletes is a currently extremely unexplored group in exercise science. This should have implications for future research, considering the possibility that effects of exercise may be dependent on sex in children (Lazaar et al., 2007; Martínez-Vizcaíno et al., 2014). Future research on HIIT in athletes should ensure to include girls.

Since none of the analyzed studies included a control group without any kind of exercise (passive control group), it is difficult to identify the main mechanism for alterations in performance, respectively in physiological parameters between maturation and training effects since young athletes are in a dynamic time of growth and development.

## Conclusions

The present systematic review revealed a growing body of literature demonstrating an efficient application of HIIT or similar training regimes, like sprint interval training, in the training routines of young and adolescent athletes from various disciplines. Based on ESs HIIT did not show a clear superiority for increasing VO_2peak_ compared to alternative training protocols. HIIT exerted a small mean ES but considerable higher percent increase of VO_2peak_ in comparison to alternative training regimes, as well as small and large mean ESs on relevant aerobic (running performance in incremental steptests) and anaerobic (sprint running, jumping, repeated sprint ability) performance parameters. Consequently, HIIT could be a time-efficient and appropriate training tool for enhancing aerobic, as well as anaerobic, performance, while leaving enough time for improving sport specific skills, as well as technique and tactics, in the training of young and adolescent athletes.

## Author contributions

FE conceived the idea of conducting this review, he defined the inclusion criteria of studies and carried out the computerized search of the electronic data bases. He computed the effect sizes and wrote the manuscript with support from BS and HC. AA supported FE with the literature search, the calculation of effect sizes and he performed the final merge of data. BS helped essential in shaping the research and providing critical feedback. He performed extensive proofreading of the manuscript and he helped to supervise the project. All authors discussed the results and contributed to the final manuscript.

### Conflict of interest statement

The authors declare that the research was conducted in the absence of any commercial or financial relationships that could be construed as a potential conflict of interest. The reviewer KM and handling Editor declared their shared affiliation.
